# Association of Gastric Myoelectrical Activity With Ghrelin, Gastrin, and Irisin in Adults With Metabolically Healthy and Unhealthy Obesity

**DOI:** 10.3389/fphys.2022.815026

**Published:** 2022-04-25

**Authors:** Mahmoud M. A. Abulmeaty, Dara Aldisi, Ghadeer S. Aljuraiban, Ali Almajwal, Eman El Shorbagy, Yara Almuhtadi, Batool Albaran, Zaid Aldossari, Thamer Alsager, Suhail Razak, Mohammed Berika, Mohamed Al Zaben

**Affiliations:** ^1^ Community Health Sciences Department, College of Applied Medical Sciences, King Saud University, Riyadh, Saudi Arabia; ^2^ Obesity Management Unit, Medical Physiology Department, School of Medicine, Zagazig University, Zagazig, Egypt; ^3^ Rehabilitation Sciences Department, College of Applied Medical Sciences, King Saud University, Riyadh, Saudi Arabia; ^4^ Surgery Department, Sultan Bin Abdulaziz Humanitarian City, Riyadh, Saudi Arabia

**Keywords:** gastric myoelectrical activity, ghrelin, gastrin, irisin, obesity phenotypes

## Abstract

**Background and Objective:** Functional disturbances of gastric myoelectrical activity (GMA) might exist in obesity. However, studies on its association with the gastric hormones in obesity phenotypes are lacking. The objective was to study the association of GMA with the serum levels of key gastric hormones in different obesity phenotypes.

**Methods:** A total of 139 adults (31.00 ± 11.12 years) were classified into different metabolic phenotypes of obesity: 1) normal weight-lean (NWL group): BMI <25 kg/m^2^ and the fat-mass index (FMI) ≤9.7 kg/m^2^ in females and ≤6.3 kg/m^2^ in males; 2) metabolically obese normal weight (MONW group): BMI <25 kg/m^2^ and FMI >9.7 kg/m^2^ in females and >6.3 kg/m^2^ in males; 3) metabolically healthy obese (MHO group): BMI ≥25 and FMI ≤9.7 kg/m^2^ in females and ≤6.3 kg/m^2^ in males; and 4) metabolically unhealthy obese (MUO group): BMI ≥25 and FMI >9.7 kg/m^2^ in females and >6.3 kg/m^2^ in males. The GMA was measured at the baseline and post-prandial state using a multichannel electrogastrography with a water load satiety test. The average power distribution by the frequency region and the average dominant frequency were used for analysis. Anthropometric measurements and bioelectric impedance analysis were performed to calculate the FMI and fat-free mass index (FFMI). Serum levels of ghrelin, gastrin, and irisin were measured by ELISA kits according to the manufacturer’s protocol.

**Results:** Compared to the NWL group, gastrin and ghrelin levels were significantly low in the MUO participants, while irisin was significantly high. The EGG showed significantly lower baseline and 20-min normogastria frequencies in the MHO and MUO groups. In the MHO group, baseline duodenal frequency was positively correlated with the gastrin level, while normogastria times were positively associated with the irisin level and negatively associated with the ghrelin level. In the MUO group, percentages of bradygastria frequencies at 10, 20, and 30 min were positively correlated with the BMI and FFMI. This bradygastria was correlated positively with the irisin level and negatively with the ghrelin level.

**Conclusion:** The EGG patterns might be associated with obesity-related gastric hormones in different obesity phenotypes. EGG may be a promising clinical tool in obesity assessment. The association of the EGG patterns with hormonal levels needs further investigation for potential practical uses.

## Introduction

Obesity has been linked with several health conditions and chronic diseases. The Global Burden of Diseases (GBD) 2015 collaborators reported that obesity is widely prevalent in the Eastern Mediterranean Region (EMR), and an increase from 15% in 1980 to 21% in 2015 was reported (GBD 2015 Eastern Mediterranean Region Obesity Collaborators, 2018). More recently, Althumiri et al. (2021) reported that the current weighted prevalence of obesity in Saudi Arabia is 24.7%. Most obese individuals have a higher intake of food than their body requirements in terms of calories (Althumiri et al., 2021). Since it is known that the gastrointestinal (GIT) system is responsible for the absorption of all nutrients, more studies have focused on the GIT involvement in the pathogenesis of obesity (Camilleri et al., 2017; Weimer et al., 2018; Farhadipour and Depoortere, 2021; van Son et al., 2021). Research has revealed that there is a high association between obesity and gastrointestinal complications (Phatak and Pashankar, 2014; Weimer et al., 2018).

Electrogastrography (EGG) is a non-invasive method used to monitor the gastric myoelectrical activity (GMA) by placing cutaneous electrodes on the abdomen. Using EGG, Weimer et al. (2018) observed impaired gastric myoelectrical reactivity in obese children compared to controls in response to the stress and water load test. Riezzo et al. (1998) observed the post-prandial pattern in response to a mixed meal in obese and normal children and found similar responses. Another study found an increase in the percentage of stomach bradyarrhythmia in both the fasting and fed states in adult individuals with morbid obesity. McCallum et al. (2001) discovered an increase in the percentage of bradygastria in both the fasting and fed states while studying adult individuals with morbid obesity.

Based on phenotypes of obesity, metabolic, and systemic consequences, obesity might start even in normal-weighted individuals. Besides BMI, the new classification of obesity considers other obesity stigmata such as fat mass and cardiovascular risk factors (De Lorenzo et al., 2020). Gut motility, gut regulatory peptides, and hormones, as well as autonomic and enteric nervous systems, all work together to keep the gastrointestinal system in good working order. These processes affect the pace at which nutrients are digested in the intestines and have all been linked to appetite and hunger regulation. The alteration of the body energy balance leads to the evolution of obesity which is associated with fat accumulation in adipose tissue (Ng et al., 2014; Adamska-Patruno et al., 2018). Being an endocrine organ, adipose tissue produces many adipokines (Khan and Joseph, 2014) that are involved in regulating energy balance and other processes (Guerre-Millo, 2004). The gastric hormones, such as ghrelin, irisin, and gastrin that have variable effects on energy balance, play an important role in the hormonal regulation of food intake (Zwirska-Korczala et al., 2007; Barja-Fernández et al., 2016; Verdeş et al., 2017; Adamska-Patruno et al., 2018; Lee et al., 2019). Zwirska-Korczala et al. (2007) in their study of women with moderate and morbid obesity and metabolic syndrome observed that plasma total and acylated ghrelin levels were significantly lower in women with obesity and metabolic syndrome than those in women with normal BMI. Despite being frequently reported as a myokine, irisin expression and secretion were proved by the chief cells of the gastric mucosa (Barja-Fernández et al., 2016). Lee et al. (2019) also reported a negative association between irisin and BMI with a 10% increase in circulating irisin concentration after 27 kg of total fat mass loss following bariatric surgery. Authors also reported a decreased but non-significant concentration of gastrin in lean women as compared to obese and morbidly obese women with a metabolic syndrome.

The current working model hypothesizes that gastric myoelectrical activity might be associated with the main gastric hormones, such as ghrelin, irisin, and gastrin, in different obesity phenotypes. This might be a mechanism that may be involved in the modification of appetite and the development of obesity secondary to changes in GMA. Consequently, different metabolic consequences of obesity will take place. Taken together, this study investigated the association of GMA with the serum levels of key gastric hormones involved in the regulation of food intake in different obesity phenotypes.

## Materials and Methods

### Study Subjects

A total of 139 adults (31.00 ± 11.12 years) from the Clinical Nutrition Clinic, College of Applied Medical Sciences at King Saud University, Saudi Arabia, were included in a case–control study between March and September 2021. About 56 non-obese individuals (BMI <29.90 kg/m^2^) and 83 patients with obesity (BMI >30.00 kg/m^2^) were reclassified according to the new phenotypic classification (De Lorenzo et al., 2016) into 1) normal weight-lean (NWL group; *n* = 17): BMI <25 kg/m^2^, and the fat-mass index (FMI) was less than the cut-off point that was defined for detecting cardiometabolic risk in the Saudi population using the atherosclerotic cardiovascular disease risk (lifetime ASCVD risk) (Abulmeaty et al., 2017), i.e., FMI ≤9.7 kg/m^2^ in females and ≤6.3 kg/m^2^ in males; 2) metabolically obese normal weight (MONW group; *n* = 8): BMI< 25 kg/m^2^ and FMI >9.7 kg/m^2^ in females and >6.3 kg/m^2^ in males, in addition to HDL ≤50 mg/dl and/or LDL ≥100 mg/dl; 3) metabolically healthy obese (MHO group; *n* = 81): BMI ≥25, FMI ≤9.7 kg/m^2^ in females and ≤6.3 kg/m^2^ in males, HDL >50 mg/dl, and LDL<100 mg/dl); and 4) metabolically unhealthy obese (MUO group; *n* = 33): BMI ≥25 and FMI >9.7 kg/m^2^ in females and >6.3 kg/m^2^ in males, in addition to HDL ≤50 mg/dl and/or LDL ≥100 mg/dl. All participants were free of any gastric problems, were not taking any medications affecting gastric motility, never underwent gastrointestinal surgeries, and were free of any acute medical conditions. The study outlines were explained to all participants before signing informed written consent. The study protocol was reviewed and approved by the IRB committee of the College of Medicine, King Saud University, under reference number 20/0908/IRB, dated: 30/11/2020.

### Sample Size Calculation

The sample size was calculated using G*Power software (G*Power 3.1.9.4, Heinrich Heine University, Dusseldorf, Germany). Based on our pilot study, correlations of the fat-mass index (FMI) and percentages of bradygastria at 30-min post-prandial periods were 0.306 and 0.652 in the control and case groups, respectively. The total sample size of 31 participants in each group was calculated based on an average two-tailed correlation of 0.48, 80% power, and α = 0.05.

### Gastric Myoelectrical Activity

The gastric myoelectrical activity was measured in fasting (for 10 min) and post-prandial states (for 30 min) using a multichannel electrogastrography (EGG) with a water load satiety test (3CPM-EGG, Sparks, MD, USA) (Wang et al., 2003). Before attending the testing session, all participants were instructed to fast for 10–12 h. Screening of gastrointestinal (GIT) symptoms and history of GIT diseases were completed before EGG recording. The recording session was carried out in a quiet room with dim-light illumination while the participant laid flat in a comfortable examination bed. Moreover, they were instructed to avoid sleeping and any body movement during the test. A respiratory belt with a sensor that records the breathing waves was wrapped around the chest above the nipple line. Then, the three disposable EGG electrodes were applied to the skin at the midline between xiphisternum and umbilicus (black electrode) and 2 inches below costal cartilage at the midclavicular line at the right side (green electrode) and the left side (red electrode) (Sharma et al., 2015). Excessive body hair present at electrode placement sites was removed before electrode application. After 10 min of pre-prandial recording, participants were instructed to drink as much plain water as possible until feeling satiety. The volume of water loading was determined and inserted into EGG software, and then the EGG recorded was resumed for 30 min after the water loading. Every 10 min, the respiratory rate was double-checked and inserted into EGG software. Selection of at least four good minutes from each 10-minute-recording interval was carried out. Based on the distribution of average power by the frequency region, bradygastria was considered if the rhythm was equal to 1.0–2.5 cycles per minute (cpm), normogastria if 2.5–3.75 cpm, tachygastria if 3.75–10.0 cpm, and duodenal respiration if 10.0–15.0 cpm. Distribution of average power by the frequency region (as a percentage of power in the 0–15 cpm range) and the average dominant frequency (ADF) in each 10-min-recording period were used for analysis.

### Anthropometric, Dietary Assessment, and Body Composition Measurement

The weight, height, and body mass index were taken as usually reported (Abulmeaty et al., 2016). The body composition was analyzed using a multi-frequency segmental bioelectric impedance analysis monitor (Tanita BC-418, Tanita Corp., Tokyo, Japan). Percent body fat (PBF), fat mass (FM), fat-mass index (FMI = FM/height^2^), fat-free mass (FFM), fat-free mass index (FFMI = FFM/height^2^) (Abulmeaty et al., 2017), visceral fat rating, and total body water (TBW) were used for analysis. Appendicular skeletal muscle mass (ASM) was calculated by the sum of the segmental muscle mass of the legs and arms. The appendicular muscle mass index (ASMI) is further calculated by ASM/height^2^ (Chang et al., 2013). Muscle function was measured from the dominant hand using a handgrip dynamometer (Jamar Hydraulic Hand Dynamometer, Warrenville, IL, USA). An average of two measures was used for the analysis (Iconaru and Ciucurel, 2020). Self-reported duration spent for physical activity was recorded. In addition, multiple passes 224-h recall for 2 days were analyzed by food processor software (ESHA Research, OR, USA). Total energy intake as total calories per day and macronutrient components were analyzed.

### Biochemical and Hormonal Analysis

Fasting blood samples were withdrawn from the antecubital vein. A complete blood count was performed within 1 h, and the serum was freshly obtained after centrifugation for 20,000 rpm for 10 min. The serum was aliquoted into four Eppendorf tubes, of which one was used for biochemical parameters and three were stored at -80 C for hormonal analysis. The biochemical parameters included a complete blood count with a differential neutrophil count, lymphocytic count, and neutrophil-to-lymphocyte ratio (Sysmex XP 300, Tokyo, Japan). The Albumin level, creatinine level, and lipid panel were measured (FUJI DRI-CHEM NX500, Japan). Serum levels of ghrelin, gastrin, and irisin were measured by ELISA kits according to the manufacturer’s protocol (MyBiosource, San Diego, CA, USA; Catalog numbers: MBS2700428, MBS264399, and MBS2600406, respectively).

### Statistical Analysis

Sociodemographic characteristics, body composition indicators, biochemical data, and gastric myoelectrical activity were compared between study groups employing one-way ANOVA (to detect overall significant differences in the mean between study groups) and Tukey’s HSD test for post hoc analysis (to compare all possible pairs of means) with Bonferroni correction or Chi-square (to compare frequencies). The Shapiro–Wilk test was used to test the normality of study variables. The relationships between body composition indicators and gastric myoelectrical activity and between gut hormones and gastric myoelectrical activity were identified using Pearson partial correlations for each study group separately. *p*-values <0.05 were considered statistically significant. SPSS software version 25 (SPSS Inc., Chicago, IL, US) was used for all analyses.

## Results

### Sociodemographic Characteristics

Approximately 35.3% of the NWL, 25% of the MONW, 60.5% of the MHO, and 60.6% of the MUO participants were women (Supplementary Table S1). Compared to participants in the NWL group, metabolically healthy and unhealthy obese participants were old ((mean [SD]); 32.7[11.5] and 32.2[11.1] *vs*. 26.2[8.5] years) and had a high income and a high lowest adult body weight (Supplementary Table S1).

### Body Composition Indicators and Other Variables

A one-way ANOVA was performed to compare the body composition indicators (BMI, fat mass, FMI, visceral fat rating, FFM, FFMI, muscle mass, and TBW) of the study groups (Table 1). The results revealed that there was an overall statistically significant difference in the group mean (BMI, fat mass, FMI, visceral fat rating, FFM, FFMI, muscle mass, TBW, basal metabolic, appendicular skeletal mass index, and average handgrip) between at least two groups (*p* < 0.0001). In addition, the duration spent on physical activity was insignificantly different among the study groups. The total caloric intake, calories from fat, and amount of carbohydrates were significantly different among the study groups (Table 1).

**TABLE 1:** Body composition indicators and other data stratified by obesity phenotypes^a^.

	Normal weight-lean (NWL)		Metabolically obese normal weight (MONW)		Metabolically healthy obese (MHO)		Metabolically unhealthy obese (MUO)		*p*-value
	Mean	(SD)	Mean	(SD)	Mean	(SD)	Mean	(SD)	
*n*	17		8		81		33		
BMI (kg/m^2^)	22.2^a^	(2.0)	21.2^a^	(1.3)	35.1^b^	(7.1)	37.7^c^	(6.9)	<0.0001
Fat (%)	26.1^a^	(6.1)	21.0^a^	(8.8)	38.0^b^	(9.2)	38.2^b^	(8.0)	<0.0001
Fat mass (kg)	16.2^a^	(4.7)	12.0^a^	(5.0)	38.2^b^	(15.5)	41.7^b^	(12.4)	<0.0001
Fat-mass index (kg/m^2^)	5.8^a^	(1.6)	4.5^a^	(2.1)	13.7^b^	(5.6)	14.8^b^	(5.3)	<0.0001
Visceral fat rating	3.4^a^	(2.6)	1.9^a^	(0.6)	12.0^b^	(6.3)	13.5^b^	(5.3)	<0.0001
Fat-free mass (kg)	45.9^a^	(8.7)	45.2^a^	(6.4)	60.2^b^	(12.5)	66.7^c^	(12.6)	<0.0001
Fat-free mass index (kg/m^2^)	16.4^a^	(1.9)	16.7^a^	(1.3)	21.4^b^	(2.9)	23.0^c^	(3.0)	<0.0001
Muscle mass (kg)	43.8^a^	(8.5)	43.1^a^	(6.2)	57.3^b^	(12.0)	63.6^c^	(12.1)	<0.0001
Total body water (kg)	33.6^a^	(6.4)	33.1^a^	(4.7)	44.3^b^	(9.4)	48.9^b^	(9.2)	<0.0001
Basal metabolic rate (kcal)	1407.5^a^	(232.6)	1384.3^a^	(164.3)	1867.4^b^	(380.9)	2070.5^c^	(379.8)	<0.0001
Appendicular skeletal muscle mass (kg)	19.5^a^	(4.7)	18 0.9^a^	(3.6)	27.0^b^	(6.9)	29.5^b^	(6.9)	<0.0001
Appendicular skeletal muscle mass index (kg/m^2^)	7.0^a^	(1.1)	5.2^b^	(3.3)	8.9^c^	(2.8)	7.8^c^	(4.5)	<0.0001
Average handgrip	23.8^a^	(4.1)	28.1^a^	(10.1)	31.8^b^	(9.5)	35.0^b^	(13.9)	0.011
Duration of physical activity (minutes/d)	28.7^a^	(26.1)	26.3^a^	(29.7)	26.7^a^	(38.7)	23.8^a^	(34.9)	0.97
Total energy (kcal/d)	1379.5^a^	(377.9)	1408.4^a^	(353.9)	1704.9^a^	(582.7)	2073.2^b^	(651.0)	0.001
Calories from fat (kcal/d)	434.1^a^	(161.6)	554.1^a^	(118.8)	532.7^a^	(215.3)	657.1^b^	(260.9)	0.016
Calories from saturated fat (kcal/d)	146.7^a^	(79.4)	151.9^a^	(46.7)	167.4^a^	(83.5)	212.6^a^	(87.5)	0.055
Amount of dietary protein (g)	69.5^a^	(41.5)	64.3^a^	(29.2)	78.1^a^	(35.2)	98.6^a^	(51.6)	0.060
Amount of dietary CHO (g)	159.6^a^	(45.7)	150.1^a^	(61.2)	203.0^a^	(72.8)	244.5^b^	(86.8)	0.001
Amount of added sugars (g)	18.1^a^	(17.9)	17.1^a^	(21.1)	21.6^a^	(19.3)	22.3^a^	(31.0)	0.885

a Values with different superscripts within a row are statistically significant (*p* < 0.05) as analyzed by ANOVA and Tukey’s HSD test for post hoc analysis with Bonferroni correction.

BMI, body mass index; CHO, carbohydrates.

Compared to those in the NWL group, mean values of BMI, fat mass, FMI, visceral fat rating, FFM, FFMI, muscle mass, TBW, basal metabolic, appendicular skeletal mass, and average handgrip were significantly high in the MHO and MUO groups (*p* < 0.05). Furthermore, the MUO group showed significantly higher BMI, FFM, FFMI, muscle mass, and BMR than the MHO group. Compared to those in the NWL group, participants in the MUO group had significantly high total calories per day, and more calories from fat, and more amount of carbohydrates than those in the NWL and MHO groups (Table 1).

### Biochemical and Hormonal Data

Parameters of the lipid panel were compared between obesity phenotype groups (Table 2). The results showed that there was an overall statistically significant difference in mean total cholesterol, HDL, LDL, creatinine, and albumin between at least two groups (*p* < 0.05). Post hoc analysis showed that the mean values of total cholesterol, LDL, and HDL were significantly different between the MUO and MHO groups (*p* < 0.05).

**TABLE 2:** Biochemical data stratified by obesity phenotypes^a^.

	Normal weight-lean (NWL)		Metabolically obese normal weight (MONW)		Metabolically healthy obese (MHO)		Metabolically unhealthy obese (MUO)		*p*-value
	Mean	(SD)	Mean	(SD)	Mean	(SD)	Mean	(SD)	
*n*	17		8		81		33		
Glucose (mg/dl)	80.0^a^	(10.9)	72.9^a^	(7.2)	84.4^a^	(27.6)	91.0^a^	(19.9)	0.177
Total cholesterol (mg/dl)	154.5^a^	(51.6)	185.0^a^	(11.6)	145.1^a^	(45.2)	195.7^b^	(26.8)	**<0.0001**
HDL (mg/dl)	50.8^a^	(14.6)	43.4^a^	(4.3)	45.2^a^	(13.1)	40.4^b^	(6.2)	**0.027**
LDL (mg/dl)	88.3^a^	(41.1)	122.1^a^	(13.7)	82.5^a,b^	(34.6)	134.1^c^	(22.3)	**<0.0001**
Triglycerides (mg/dl)	77.1^a^	(33.0)	97.4^a^	(48.2)	87.2^a^	(49.7)	106.0^a^	(48.7)	**0.152**
Creatinine (mg/dl)	0.7^a^	(0.2)	1.8 ^b^	(2.9)	0.7^a^	(0.2)	0.8^a^	(0.2)	**<0.0001**
Albumin (g/dl)	4.1^a^	(0.9)	4.5^a^	(0.6)	4.0^a^	(0.8)	4.5^a^	(0.6)	**0.044**
RBC (10^b^3/uL)	4.9^a^	(0.5)	5.1^a^	(0.6)	5.2^a^	(0.6)	5.2^a^	(0.5)	0.340
Hct (%)	39.6^a^	(3.9)	42.6^a^	(5.2)	41.8^a^	(4.1)	42.3^a^	(4.3)	0.153
Hb (g/dl)	13.7^a^	(1.7)	15.1^a^	(1.8)	14.6^a^	(1.8)	14.6^a^	(1.8)	0.206
MCH (pg)	28.0^a^	(3.1)	29.6^a^	(1.2)	28.4^a^	(2.6)	28.2^a^	(2.5)	0.497
MCV (fL)	80.9^a^	(5.7)	83.8^a^	(2.8)	81.3^a^	(5.0)	81.7^a^	(5.4)	0.579
WBC (10^b^3/uL)	5.6^a^	(1.0)	7.0^a^	(1.8)	6.6^a^	(1.9)	6.8^a^	(1.6)	0.084
Neutrophils (10^b^3/uL)	2.3^a^	(1.2)	4.2^b^	(1.9)	3.8^b^	(1.5)	4.1^b^	(1.5)	**0.008**
Lymphocytes (10^b^3/uL)	1.57^a^	(1.0)	2.24^a^	(0.54)	2.1^a^	(0.8)	2.1^a^	(0.7)	0.234
Neutrophil-to-lymphocyte ratio	2.9^a^	(2.8)	2.1^a^	(1.15)	2.4^a^	(1.9)	2.5^a^	(1.6)	0.799
Platelets (10^b^3/uL)	250.7^a^	(63.7)	217.8^a^	(64.1)	271.8^a^	(73.7)	289.4^a^	(95.1)	**0.086**
Gastrin (pg/ml)^b^	56.3^a^	(7.2)	54.2^a^	(6.7)	42.1^a^	(31.8)	31.3^b^	(20.4)	**0.035**
Ghrelin (pg/ml)^b^	366.9^a^	(33.8)	370.6^a^	(38.5)	179.4^b^	(93.4)	140.4^b^	(59.1)	**<0.0001**
Irisin (ng/ml)^b^	8.3^a^	(1.5)	8.6^a^	(1.4)	19.7^b^	(9.2)	19.0^b^	(7.6)	**<0.0001**

a Values with different superscripts within a row are statistically significant (*p* < 0.05) as analyzed by ANOVA and Tukey’s HSD test for post hoc analysis with Bonferroni correction.

b Analyzed in a subsample (*n* = 60).

Hb, hemoglobin; Hct, hematocrit; HDL, high-density lipoprotein; LDL, low-density lipoprotein; MCH, mean corpuscular hemoglobin; MCV, mean corpuscular volume; RBC, red blood cell; WBC, white blood cell.

Bold values means statistically significant.

The mean neutrophil count was significantly different between at least two groups (*p* < 0.05). Post hoc analysis showed that neutrophils were lower in the NWL group than in MONW, MHO, and MUO groups. The lymphocyte level and NLR were insignificantly different among the study groups (*p* > 0.05).

There was an overall statistically significant difference in mean gastrin, ghrelin, and irisin levels among the study groups (*p* < 0.05). Compared to those in the NWL group, gastrin and ghrelin levels were significantly lower in the MUO participants (31.3 [20.4] *vs*. 56.3 [7.2] pg/ml and (140.4 [59.1] *vs*. 366.9 [33.8] ng/ml, respectively), while irisin was significantly high (19.0 [7.6] *vs*. 8.3 [1.5] pg/ml). Moreover, the means of ghrelin and irisin in the MUO group were insignificantly different from those in the MHO group (*p* > 0.05), while gastrin showed significantly lower values (31.3 [20.4] *vs*. 42.1 [31.8] pg/ml) (Table 2).

### Gastric Myoelectrical Activity Using Electrogastrography

The pre-prandial gastric myoelectrical activity was compared among the study groups and showed an overall statistically significant difference in mean baseline normogastria frequencies (*p* < 0.0001). Compared to that of the NWL group, the percentage of normogastria times was significantly low in the MHO group (*p* < 0.05) with a further significant reduction in the MUO group (Table 3).

**TABLE 3:** Gastric myoelectrical activity using electrogastrography (EGG), stratified by obesity phenotypes^a^.

	Normal weight-lean (NWL)		Metabolically obese normal weight (MONW)		Metabolically healthy obese (MHO)		Metabolically unhealthy obese (MUO)		*p*-value
	Mean	(SD)	Mean	(SD)	Mean	(SD)	Mean	(SD)	
*n*	17		8		81		33		
Baseline bradygastria (%)	53.4^a^	(22.6)	41.7^a^	(10.3)	54.7^a^	(20.0)	53.0^a^	(27.5)	0.464
Baseline normogastria (%)	22.0^a^	(20.4)	28.1^a^	(16.1)	14.1 ^b^	(8.7)	10.6^c^	(7.6)	**<0.0001**
Baseline tachygastria (%)	16.9^a^	(8.5)	20.3^a^	(6.5)	19.9^a^	(13.8)	21.1^a^	(14.7)	0.753
Baseline duodenal (%)	7.8^a^	(7.1)	9.8^a^	(6.5)	11.4^a^	(12.5)	15.3^a^	(18.1)	0.268
10 min bradygastria (%)	56.3^a^	(17.5)	63.4^a^	(19.2)	57.3^a^	(18.7)	54.8^a^	(20.7)	0.713
10 min normogastria (%)	15.7^a^	(8.9)	12.4^a^	(7.7)	14.0^a^	(8.3)	10.6^a^	(6.6)	0.113
10 min tachygastria (%)	20.1^a^	(13.3)	18.3^a^	(13.0)	20.9^a^	(12.2)	21.9^a^	(11.5)	0.883
10 min duodenal (%)	8.0^a^	(8.8)	5.8^a^	(5.3)	7.8^a^	(8.6)	12.7^a^	(14.5)	0.101
20 min bradygastria (%)	51.1^a^	(22.1)	43.7^a^	(23.4)	51.0^a^	(17.9)	52.9^a^	(17.6)	0.666
20 min normogastria (%)	19.3^a^	(13.5)	33.4 ^b^	(23.0)	18.0^a^	(11.8)	16.1^a^	(10.2)	**0.006**
20 min tachygastria (%)	21.2^a^	(11.6)	17.9^a^	(9.2)	22.8^a^	(12.3)	18.7^a^	(7.8)	0.256
20 min duodenal (%)	8.4^a^	(9.5)	5.0^a^	(6.1)	8.1^a^	(9.5)	12.4^a^	(17.9)	0.271
30 min bradygastria (%)	49.2^a^	(19.5)	46.3^a^	(23.4)	52.5^a^	(17.7)	45.8^a^	(17.2)	0.295
30 min normogastria (%)	22.4^a^	(14.9)	22.6^a^	(18.6)	20.0^a^	(13.0)	20.4^a^	(13.0)	0.894
30 min tachygastria (%)	20.8^a^	(8.7)	23.7^a^	(15.6)	20.7^a^	(11.0)	23.2^a^	(10.8)	0.667
30 min duodenal (%)	7.7^a^	(8.6)	7.4^a^	(6.2)	6.7^a^	(6.7)	10.7^a^	(15.8)	0.282
The average dominant frequency at first 10 min	1.6^a^	(0.8)	2.0^a^	(0.8)	2.0^a^	(1.6)	2.6^a^	(3.1)	0.359
The average dominant frequency at 10–20 min	2.1^a^	(1.4)	1.6^a^	(0.4)	1.9^a^	(1.8)	2.3^a^	(2.8)	0.712
The average dominant frequency at 20–30 min	2.7^a^	(2.6)	2.1^a^	(0.9)	1.9^a^	(1.3)	2.2^a^	(2.5)	0.434
The average dominant frequency at 30–40 min	1.6^a^	(1.0)	1.8^a^	(1.1)	1.5^a^	(1.0)	2.2^a^	(3.3)	0.391
Water load (ml)	543.5^a^	(186.0)	455.0^a^	(165.0)	555.3^a^	(285.8)	618.3^a^	(305.5)	0.443

a Values with different superscripts within a row are statistically significant (*p* < 0.05) as analyzed by ANOVA and Tukey’s HSD test for post hoc analysis with Bonferroni correction.

Bold values means statistically significant.

At 10–20 min of the post-prandial recording, the percentages of normogastria frequencies showed an overall statistically significant difference across the study groups. Multiple comparisons showed significantly higher percentages of normogastria frequencies in the MONW group than those in the NWL group (*p* < 0.05) (Table 3). Other EGG parameters were insignificantly different among the study groups.

### Association Between Body Composition Indicators and Gastric Hormones, With Gastric Myoelectrical Activity Stratified by the Obesity Phenotype

Pearson partial correlations between body composition indicators and gastric myoelectrical activity were analyzed separately in each study group. In the NWL group, the correlation coefficient between BMI and normogastria at 10-min was inverse (Table 4). Later in the post-prandial recording, BMI showed a negative correlation with 30-min bradygastria and positive correlations with 30-min normogastria and duodenal rhythm, in addition to a positive correlation with the volume of water load (Table 4). The FMI was not significantly correlated with any indices. The FFMI showed a negative correlation with baseline and 10-min normogastria percentages and a positive correlation with 10-min bradygastria. At 30-min, FFMI was correlated positively with normogastria percentages and negatively with bradygastria percentages (Table 4). Furthermore, no significant correlations of gastrin and irisin hormones with the gastric myoelectrical activity were observed in the NWL group. However, ghrelin showed positive correlations with the average dominant frequency at 10–20 min and percentage of 10-min duodenal frequency (Table 5).

**TABLE 4:** Pearson partial correlations between body composition indicators and gastric myoelectrical activity in each group of obesity phenotypes.

	Normal weight-lean			Metabolically obese normal weight			Metabolically healthy obese			Metabolically unhealthy obese		
	BMI	Fat-mass index	Fat-free mass index	BMI	Fat-mass index	Fat-free mass index	BMI	Fat-mass index	Fat-free mass index	BMI	Fat-mass index	Fat-free mass index
Baseline bradygastria	0.01	−0.35	0.29	0.55	0.63	−0.44	0.21	0.20	0.12	0.25	0.07	0.43**
Baseline normogastria	−0.33	0.21	−0.52*	-0.14	−0.18	0.14	0.06	0.13	−0.11	−0.04	−0.03	−0.05
Baseline tachygastria	0.42	0.34	0.16	0.08	−0.28	0.52	-0.05	-0.07	0.00	−0.04	0.10	−0.29
Baseline duodenal	0.45	0.11	0.38	−0.61	−0.28	−0.18	−0.31**	−0.33**	−0.12	−0.32	−0.19	−0.40*
10 min bradygastria	0.32	−0.31	0.60**	0.34	0.36	−0.23	0.09	0.09	0.04	0.40*	0.24	0.47*
10 min normogastria	−0.62**	−0.14	−0.54*	0.11	0.26	−0.31	0.04	0.07	-0.04	0.02	0.00	0.06
10 min tachygastria	0.02	0.40	−0.30	−0.38	−0.46	0.34	−0.09	−0.12	−0.01	−0.35	−0.18	−0.48**
10 min duodenal	−0.05	0.15	−0.17	−0.45	−0.57	0.45	−0.10	−0.11	-0.03	−0.30	−0.20	−0.32
20 min bradygastria	−0.11	−0.08	−0.05	0.46	0.23	0.11	0.02	0.02	0.02	0.40*	0.25	0.45**
20 min normogastria	0.08	0.08	0.02	−0.27	−0.06	−0.17	0.04	0.05	0.01	−0.06	0.03	−0.17
20 min tachygastria	0.03	0.12	−0.06	−0.62	−0.38	−0.04	−0.05	−0.04	−0.04	−0.15	−0.11	−0.13
20 min duodenal	0.10	−0.05	0.15	0.19	−0.07	0.30	−0.02	−0.03	0.01	−0.29	−0.21	−0.29
30 min bradygastria	-0.70***	−0.21	−0.56*	0.56	0.59	−0.36	0.15	0.20	−0.02	0.62***	0.46**	0.60***
30 min normogastria	0.53*	0.08	0.49*	−0.08	0.03	−0.12	0.02	−0.01	0.06	−0.21	−0.18	−0.17
30 min tachygastria	0.14	−0.07	0.20	−0.51	−0.70	0.59	−0.20	−0.22*	−0.06	−0.22	−0.13	−0.26
30 min duodenal	0.53*	0.42	0.21	−0.61	−0.54	0.25	−0.10	−0.14	0.03	−0.35*	−0.27	−0.31
The average dominant frequency at first 10 min	−0.32	0.12	−0.44	−0.52	−0.73*	0.64	−0.16	−0.12	−0.14	−0.29	−0.09	−0.49**
The average dominant frequency at 10–20 min	−0.29	0.02	−0.31	0.09	0.01	0.07	−0.04	−0.01	−0.08	−0.27	−0.16	−0.34
The average dominant frequency at 20–30 min	0.41	0.27	0.21	−0.51	−0.24	−0.14	−0.08	−0.12	0.03	−0.24	−0.13	−0.31
The average dominant frequency at 30–40 min	0.32	0.10	0.26	−0.25	−0.15	−0.01	−0.24*	−0.24*	−0.14	−0.29	−0.17	−0.35*
Water load	0.53*	0.31	0.31	−0.26	−0.41	0.39	0.11	0.04	0.20	0.02	−0.17	0.34*

*significance at *p* < 0.05, **significance at *p* < 0.01, and ***significance at *p* < 0.001.

**TABLE 5:** Pearson partial correlation between gut hormones and gastric myoelectrical activity in each group of obesity phenotypes.

	Normal weight-lean			Metabolically obese normal weight			Metabolically healthy obese			Metabolically unhealthy obese		
	Gastrin	Ghrelin	Irisin	Gastrin	Ghrelin	Irisin	Gastrin	Ghrelin	Irisin	Gastrin	Ghrelin	Irisin
Baseline bradygastria	−0.38	0.17	0.27	0.16	−0.13	−0.35	−0.32	−0.11	0.09	0.05	0.10	−0.18
Baseline normogastria	0.53	0.19	−0.21	−.54	0.56	−0.03	−0.13	−0.57**	0.45*	−0.13	−0.22	0.27
Baseline tachygastria	−0.20	−0.53	−0.06	0.50	−0.71	0.52	−0.04	0.05	−0.04	−0.03	−0.26	0.39
Baseline duodenal	−0.16	−0.38	−0.11	0.60	−0.46	0.12	0.53**	0.34	−0.26	−0.01	0.09	−0.08
10 min bradygastria	−0.37	−0.51	−0.03	−0.29	−0.27	0.59	−0.05	−0.13	0.17	−0.08	−0.17	0.12
10 min normogastria	0.01	0.01	−0.19	0.02	0.95***	−0.08	0.02	−0.27	0.20	−0.32	−0.09	0.09
10 min tachygastria	0.29	0.00	0.10	0.23	−0.05	−0.65	−0.03	0.16	−0.15	0.25	0.26	−0.20
10 min duodenal	0.27	0.82**	0.12	0.46	−0.27	−0.42	0.14	0.23	−0.28	0.08	0.11	−0.08
20 min bradygastria	0.07	0.24	0.01	0.24	−0.29	−0.07	0.07	−0.11	0.14	−0.40	−0.25	0.23
20 min normogastria	−0.07	−0.32	−0.06	−0.37	0.63	0.08	0.05	−0.24	0.25	0.46	0.06	−0.10
20 min tachygastria	0.13	0.10	0.16	0.18	−0.56	−0.16	−0.09	0.26	−0.24	0.18	−0.01	−0.05
20 min duodenal	−0.24	−0.13	−0.10	0.21	−0.41	0.21	−0.07	0.11	−0.21	0.08	0.19	−0.14
30 min bradygastria	−0.13	0.08	0.24	−0.31	−0.42	−0.06	0.00	−0.11	−0.08	−0.32	−0.69**	0.69**
30 min normogastria	−0.11	−0.13	−0.25	0.02	0.87**	0.13	0.04	−0.12	0.39	0.35	0.32	−0.28
30 min tachygastria	0.36	0.20	0.09	0.42	−0.28	0.16	−0.02	0.19	−0.10	−0.16	0.20	−0.33
30 min duodenal	0.16	−0.12	−0.11	0.02	−0.30	−0.56	−0.01	0.18	−0.23	0.11	0.31	−0.26
The average dominant frequency at first 10 min	0.00	−.34	−0.26	−0.16	0.06	−0.04	0.29	−0.01	0.07	−0.03	0.04	−0.08
The average dominant frequency at 10–20 min	0.45	0.82**	0.10	0.53	−0.31	0.58	−0.09	0.04	−0.14	0.14	0.28	−0.22
The average dominant frequency at 20–30 min	−0.06	−0.38	−0.19	−0.37	0.20	0.12	−0.11	0.13	−0.15	0.21	0.29	−0.23
The average dominant frequency at 30–40 min	0.12	−0.20	−0.27	0.18	0.38	0.41	−0.05	0.22	−0.05	0.23	0.39	−0.32
Water load	0.42	−0.07	−0.18	0.32	−0.75*	0.55	−0.08	0.06	0.09	−0.07	0.07	0.08

*significance at *p* < 0.05, **significance at *p* < 0.01, and ***significance at *p* < 0.001.

In the MONW group, BMI and FFMI were not significantly correlated, while the FMI was inversely correlated with the average dominant frequency at 10–20 min (Table 4). The ghrelin level showed a positive correlation with normogastria frequency at 10 and 30 min and an inverse correlation with the volume of water load (Table 5).

As for the MHO group, the baseline duodenal frequency and the average dominant frequency at 30–40 min showed significant inverse correlations with BMI and FMI (Table 4). Moreover, the baseline duodenal frequency was positively correlated with the gastrin level. The baseline normogastria level was positively correlated with the irisin level and negatively with the ghrelin level (Table 5).

In the MUO group, percentages of bradygastria frequencies at 10, 20, and 30 min were correlated positively with the BMI (r = 0.40, 0.40, and 0.62, respectively, P trend <0.05). Also, the FMI was positively correlated with bradygastria at 30 min (r = 0.46, *p* < 0.01) (Table 4). The FFMI showed similar associations with bradygastria frequencies at baseline, 10, and 30 min. Furthermore, FFMI correlated positively with the volume of water load and negatively with baseline duodenal frequency, 10-min tachygastria, and average dominant frequencies at 10 and 30 min. In addition, the percentage of 30-min bradygastria was positively correlated with the irisin level and negatively with the ghrelin level (Table 5).

## Discussion

This study compared body composition, biochemical parameters, gastric hormonal levels, and gastric myoelectrical activity in an adult sample stratified by the obesity metabolic phenotypes into four groups (NWL, MONW, MHO, and MUO). Compared to the NWL group, the glucose level was not significantly different among the study groups, while lipid panels were significantly different. This was relatively consistent with definitions of MHO that are usually reported in the literature (Tsatsoulis and Paschou, 2020; Bosy‐Westphal and Müller, 2021). Higher irisin levels in individuals with MHO were reported by Bonfante et al. (2017), who found that patients with obesity, in whom irisin levels were high, had better metabolic health and a lower risk of type 2 diabetes. However, the irisin level was insignificantly different between the MHO and MUO groups in the current study. Being mainly a myokine, similar means of irisin levels in both MHO and MUO groups might be due to similar appendicular skeletal muscle mass in both groups. The higher muscle mass in the MUO is not indicative of increased skeletal muscle mass because the Tanita device measures the total muscle mass including cardiac and smooth muscles with their water content. Mai et al. (2020) reported low irisin levels in adults with Prader–Will syndrome than those with regular obesity suggesting a potential link between circulating irisin and obesity-related metabolic dysfunctions. In this context, in the current study, irisin was associated with times of normogastria in the MHO group indicating a potential marker of normal GMA in this disease population. This may support the clinical value of the irisin level as an indicator of metabolic health in obesity.

The body compositional differences were as expected in the four study groups, emphasizing the progressive increase of lean compartment parameters (FFM, FFMI, muscle mass, ASM, ASMI, and handgrip power) in parallel with the increased body adiposity. In this context, despite having normal BMI, the appendicular skeletal muscle mass index was significantly lower in the MONW group than that of the NWL group. In the MUO group, the total energy intake was much higher than that in the MHO with more calories from fat and a higher amount of carbohydrates. This finding points to the possible dietary causes of obesity development. In addition, the duration spent in doing physical activity was similar in all study groups. Phillips et al. (2013) reported that total energy intake, the macronutrient composition of the diet, and physical activity level were insignificantly different between the metabolically healthy and unhealthy groups regardless of the BMI. Another opinion from China reported that relatively high-carbohydrate and low-fat intakes were associated with a higher risk of obesity development in rural Chinese adults (Zhang et al., 2022). In daily clinical practice, it may be worthy to do the dietary assessment during obesity evaluation.

The gastric myoelectrical activity showed distinctive patterns among the study groups. A pre-prandial and 20-min post-prandial EGG study showed progressive reductions in percentages of normogastria times in the MHO and MUO groups compared to the NWL group. This was inconsistent with the pre-prandial findings of previous reports in children and adolescents (Riezzo et al., 1998; Weimer et al., 2018). In contrast, Tolj et al. (2007) reported that BMI impacted pre-prandial tachygastria and arrhythmia. Also, they concluded that age was found to affect the pre-prandial EGG parameters rather than post-prandial dominant frequency, coefficient of variation for dominant frequency, and gastric arrhythmia. Moreover, Simonian et al. (2004) concluded that there is no significant difference in the EGG parameters regarding age or gender. They also found that individuals with a BMI >25 kg/m^2^ had a significant reduction in the absolute dominant frequency but a similar increase in the post-prandial dominant frequency power. However, these studies did not classify individuals with high BMI into different obesity phenotypes.

Despite similar metabolic disturbances, BMI was important in the impact of metabolic health on the GMA. In this context, participants in the MONW group showed significantly higher percentages of normogastria than those in the NWL group, while the percentages of normogastria in the MUO group were significantly lower than those in the MHO group. Tolj et al. (2007) stated that BMI might affect the post-prandial dominant frequency, coefficient of variation for dominant frequency, and gastric arrhythmia. In addition, it was reported that individuals with high BMI had a post-prandial reduction in the percentage of slow-wave coupling compared to normal-weighted controls (Simonian et al., 2004). At least in part, the changes in the fat layer in a patient with morbid obesity could affect the power of EGG tracing. Conductivity discontinuities may be produced by excessive low-conductivity adipose tissue in the abdomen (Bradshaw et al., 2001). These modifications in the conductivity of the anterior abdominal wall in morbid obesity may attenuate and/or distort the electrical potential of the gastric slow wave (Obioha et al., 2013). However, this mechanical factor may reduce the amplitude of EGG electric potential rather than the frequency, and in this study we analyzed the frequency-related parameters of the EGG. Moreover, the amplitude of the gastric waves depends on the fasting–fed-state of the stomach (Wolpert et al., 2020), while all of our participants were fasting. Both MHO and MUO groups included morbid BMI participants with similar mechanical challenges of the EGG recording. However, normogastria times were significantly lower in the MUO than those in the MHO group. This may ring a bell regarding BMI-independent factors that may affect GMA.

The changes in the EGG frequency with obesity phenotypes are associated with body composition parameters. In the NWL group, the increase of the BMI units (and similarly FFMI) was associated with EGG changes (Table 3) indicating more active and larger stomachs. The increase of ADF, especially in early post-prandial (10-min), was associated with increased plasma ghrelin levels. However, FMI failed to give any significant association in the NWL group, while in the MONW group, with the augmentation of FMI, a rise of FMI was associated with reduction of ADF at 10 min. In this obesity phenotype (despite being of normal BMI), ghrelin levels were significantly higher than those in the MHO and MUO groups (Table 2), producing orexigenic effects that may depend on hypothalamic adenosine monophosphate-activated protein kinase (AMPK) activity (Scerif et al., 2010). In the MONW group, the ghrelin level was associated with normogastria times at 10 and 30 min of post-prandial recording. This may explain why normogastria times were high in MONW and low in the MUO and MHO groups.

In the MHO group, the higher FMI/BMI, the lower ADF, and percentages of tachy-rhythms of the stomach were observed. This was further continued in the MUO group, in which the BMI/FFMI was positively correlated with the times of bradygastria at, baseline, 10, 20, and 30 min. In the MHO group, the gastrin hormone level was associated with the duodenal rhythm of the stomach. The noticed hypogastrinemia in the MUO group was consistent with the state of slowing down of gastric slow wave firing because of loss of the stimulatory role of gastrin on gastric motility (Prosapio et al., 2018). In line with this hypothesis, the study of gastric electric activity in dyspeptic patients showed more percentages of tachygastria and more gastrin levels (Riezzo et al., 2001). The current study stated a reduction of ghrelin levels in MHO and MUO groups in comparison with the NWL controls with a further insignificant reduction in the MUO group compared to the MHO group. Generally, some studies reported that the ghrelin level was lower in obese patients than that in lean individuals depending on the BMI (Makris et al., 2017) without considering metabolic phenotypes. However, some opinions stated that ghrelin levels were insignificantly different among normal-weighted, overweighed, and obese individuals (Sitar-Tˇaut et al., 2021). These previous reports depend on BMI only in group comparison without considering metabolic phenotypes. Interestingly, the ghrelin level was inversely associated with the percentage of normogastria times indicating a disturbed rhythm in states with hypoghrelinemia such as the MUO. Przybylska-Feluś et al. (2016) noticed that decreasing normogastria times are associated with a reduction in ghrelin and a rise of the pancreatic polypeptide in patients with celiac disease.

In the MUO group, positive correlations of FFMI/BMI with bradygastria times may put a basis of states of functional disorders frequently reported in patients with obesity such as reflux and dyspepsia. Interestingly, the rise of FFMI was associated with increased water load volumes indicating a lazy and large stomach. The association studies of the EGG parameters and gastric hormones in obesity are limited (McNearney et al., 2009). To the best of our knowledge, this work is the first to link the gastric hormones to the EGG parameters in different obesity metabolic phenotypes. As potential clinical application, proper modification of the gastric myoelectrical activity may lead to amelioration of GIT hormone disturbances that may predispose to obesity. Previous trials of gastric electric stimulations could support the hypothesis of “modifications of gastric electrical potentials could induce alterations of gastric hormonal levels.” Despite the variable degrees of weight loss, some studies stated a reduction in the ghrelin level (Bohdjalian et al., 2009), others stated an increase in the ghrelin level (Cigaina and Hirschberg et al., 2007), and some reported non-significant changes in the ghrelin level (Sanmiguel et al., 2007). These variable findings may be due to variations in the technology used and the electrical status of the stomach. Coupling individualized recording of the EGG and adjustment of the pacemaker based on the EGG of the patient himself might give promising results of this minimally invasive anti-obesity treatment option.

The coupling of electrical potential changes and hormonal release might be mediated *via* local and central pathways. Locally, it was reported that endocrine cells, even in the pituitary gland, have extracellular ligand-gated ion channels, and their activation leads to amplification of the pacemaking activity and induces calcium influx which leads to the release of a hormone (Stojilkovic et al., 2010). Furthermore, this excitation–secretion coupling involves the presence of G-protein-coupled receptors on the endocrine cell membrane, which can stimulate or inhibit the cellular electrical activity, action potential-dependent Ca-influx, and hormone release (Fletcher et al., 2018). The stomach as a part of GIT can be considered an electrically excitable system, and any disruption in its normal electrophysiology could produce motility disorders such as gastroparesis and gastroesophageal reflux disease. There is also a pacemaker, interstitial cells of Cajal (ICC), which generates slow-wave activity that spreads into the gastric wall (Tse et al., 2016). The ICC also contains a voltage-gated L-type Ca^2+^ channel which is activated at -60 mV in addition to mechanosensitive ion channels that respond to mechanically gated changes in ion channel currents leading to modulation of the electrical slow-wave of the ICC (Kraichely and Farrugia, 2007; Joshi et al., 2021). Centrally, brain–stomach coupling is evident in humans (Wang Y. B. et al., 2020). Simultaneous recording of the EGG in normal-weighted subjects at rest with the recording of brain activity with functional magnetic resonance imaging revealed regions where spontaneous fluctuations in the blood-oxygen-level-dependent signals were synchronized with the EGG rhythm. These regions include mid-cingulate areas, primary and secondary somatosensory cortices, and portions of the occipital lobe where food cravings could be modulated (Rebollo et al., 2018; Wang G-J. et al., 2020; Wolpert et al., 2020).

In conclusion, in the NWL controls, the EGG study showed larger and more active stomachs in those with increasing BMI and FFMI. Then, in the MHO group, general slowing of the EGG frequency was noticed and associated with gastric hormonal changes such as gastrin and ghrelin levels. Finally, in the MUO group, a large and slow stomach was evident with associated changes in the irisin serum level. EGG testing may be a promising clinical indicator during obesity assessment. The association of the EGG patterns with hormonal levels needs further investigation for potential therapeutic uses.

## Data Availability

The raw data supporting the conclusion of this article will be made available by the authors, without undue reservation.
