# Supplementary material for: Association of Gastric Myoelectrical Activity With Ghrelin, Gastrin, and Irisin in Adults With Metabolically Healthy and Unhealthy Obesity
Source: Front Physiol. 2022 Apr 25;13:815026. doi: 10.3389/fphys.2022.815026 (PMC9081643; doi:10.3389/fphys.2022.815026)
Supplement: Supplementary file 1 [file Table1.DOCX]

| **Table S1. Sociodemographic characteristics stratified by obesity phenotype^1^** | | | | | | | |  |  |
| --- | --- | --- | --- | --- | --- | --- | --- | --- | --- |
| **Variable** | **Normal weight lean** | | **Metabolically obese normal weight** | | **Metabolically healthy obese** | | **Metabolically unhealthy obese** | | **P-value** |
| *n* | 17 |  | 8 |  | 81 |  | 33 |  |  |
| **Gender** |  |  |  |  |  |  |  |  |  |
| Male | 64.7 |  | 75.0 |  | 39.5 |  | 39.4 |  | <0.0001 |
| Female | 35.3 |  | 25.0 |  | 60.5 |  | 60.6 |  |  |
| Age (y) | 26.2 | (8.5) | 22.3 | (4.9) | 32.7 | 11.5 | 32.2 | 11.1 | 0.01 |
| Age at the lowest wt (y) | 17.3 | (3.0) | 19.1 | (4.6) | 20.7 | (12.1) | 19.1 | (4.9) | 0.61 |
| **Nationality** |  |  |  |  |  |  |  |  |  |
| Saudi | 88.2 |  | 87.5 |  | 79.8 |  | 73.3 |  | 0.71 |
| Non-Saudi | 11.8 |  | 12.5 |  | 20.2 |  | 26.7 |  |  |
| **Income (SR)** |  |  |  |  |  |  |  |  |  |
| < 120K per year | 75.0 |  | 66.7 |  | 33.3 |  | 58.3 |  | 0.07 |
| 120 - 240K per year | 12.5 |  | 22.2 |  | 41.7 |  | 33.3 |  |  |
| > 240K per year | 12.5 |  | 11.1 |  | 25.0 |  | 8.4 |  |  |
| **Education** |  |  |  |  |  |  |  |  |  |
| High school | 25.0 |  | 9.1 |  | 18.2 |  | 16.7 |  | 0.73 |
| Diploma | 8.3 |  | 0.0 |  | 13.6 |  | 12.5 |  |  |
| BSc | 58.3 |  | 90.9 |  | 40.9 |  | 52.8 |  |  |
| Postgraduate | 8.4 |  | 0.0 |  | 27.3 |  | 18.0 |  |  |
| **Marital status** |  |  |  |  |  |  |  |  |  |
| Single | 81.0 |  | 50.0 |  | 42.1 |  | 46.7 |  | 0.32 |
| Married | 19.0 |  | 42.9 |  | 55.3 |  | 53.3 |  |  |
| Divorced | 0.0 |  | 7.1 |  | 2.6 |  | 0.0 |  |  |
| **History of chronic disease** |  |  |  |  |  |  |  |  |  |
| Yes | 25.0 |  | 0.0 |  | 25.0 |  | 50.0 |  | 0.23 |
| No | 75.0 |  | 100.0 |  | 75.0 |  | 50.0 |  |  |
| Lowest adult weight | 52.8 | (16.1) | 61.2 | (13.1) | 70.7 | (13.4) | 82.5 | (17.8) | <0.0001 |
| Duration of physical activity (min/d) | 48.3 | (13.2) | 69.5 | (50.2) | 55.9 | (32.5) | 40.7 | (11.7) | 0.26 |
| ^1^ Values are mean (SD) or frequency (%) | | |  |  |  |  |  |  |  |
